# Diet as a Therapeutical Measure in Diseases of the Skin

**Published:** 1907-06

**Authors:** 


### DIET AS A THERAPEUTICAL MEASURE IN DISEASES OF THE SKIN.

---

Fox emphasizes the following: The importance of a careful regulation of the diet in the treatment of all inflammatory affections of the skin, including all skin affections in which the congestive element is present in a greater or lesser degree. While dieting cannot be expected to cure lupus, syphilis, or malignant disease, it certainly can improve the condition of the patient and modify the local conditions to a slight extent, and hence may prove of service in nearly all skin diseases. The habit of eating too much. While it is undeniable that some of our patients suffer from malnutrition and need to be better nourished before their skin disease can be radically cured, it is also true that even these patients can often be benefited by a temporary restriction of diet. For every patient suffering from inflammatory skin disease who is eating too little and suffering from lack of nourishment, we meet with a score or more who eat far more than they actually need and feed the eruption for which they seek relief. In advising our patients what to eat, it is wrong to say "don't eat this or don't eat that," for one might spend an hour or more in making out an expurgated diet list, and still leave foods unmentioned which might, if taken in excess or at the wrong time, prove extremely detrimental. It is far better to name a few nutritious and easily digested articles. Limit the patient to these for a short time, stating that other foods are not necessarily injurious, but that they will certainly do no harm if they are not eaten. Such a plan enables the physician to know exactly what his patient is eating and helps him to ascertain what does good and what does harm. The fact that the character of the food advisable in the treatment of cutaneous and other diseases is of less importance, perhaps, than the manner in which this food is taken. It is not what a patient eats, so much as it is how, and when, and under what circumstances he eats it, that tends to the production of an inflammatory condition of the skin. Hasty eating, irregular eating, and meals taken under the stress of excitement and worry, are the daily experiences of many of our patients both rich and poor.—Journal Cutaneous Diseases, April, 1907.

